# Acceptance and Commitment to Empowerment Intervention to Reduce HIV Stigma and Promote Community Resilience: Protocol for an Implementation Study

**DOI:** 10.2196/80669

**Published:** 2026-01-26

**Authors:** Josephine Pui-Hing Wong, Alan Tai-Wai Li, Carla T Hilario, Mandana Vahabi, Egbe Etowa, Isaac Luginaah, Aniela M dela Cruz, Miya Narushima, Kenneth Po-Lun Fung

**Affiliations:** 1 Daphne Rockwell School of Nursing, Toronto Metropolitan University, 350 Victoria St, Toronto, ON, M5B 2K3, Canada, 1 416-979-5000; 2 Primary Care, Maple Leaf Medical Clinic, Toronto, ON, Canada; 3 School of Nursing, University of British Columbia Okanagan, Kelowna, BC, Canada; 4 Lawrence Bloomberg Faculty of Nursing, University of Toronto, Toronto, ON, Canada; 5 Department of Geography and Environment, Western University, London, ON, Canada; 6 Faculty of Nursing, University of Calgary, Calgary, AB, Canada; 7 Faculty of Applied Health Sciences, Brock University, St. Catharines, ON, Canada; 8 Department of Psychiatry, University of Toronto, Toronto, ON, Canada

**Keywords:** acceptance commitment to empowerment, HIV stigma reduction, racialized communities, train-the-trainer approach, capacity building

## Abstract

**Background:**

Racialized immigrants are disproportionately impacted by HIV in Canada. In 2022, 69.5% of first-time HIV cases, where race and/or ethnicity was reported, were among racialized individuals: Indigenous (22.6%), Black (18.0%), Asian and Arab (16.3%), and Latinx (10.1%). HIV vulnerability in racialized communities is reinforced by HIV stigma intersecting with prejudice and discrimination associated with racism, gendered oppression, homophobia, and economic marginalization. Stigma and discrimination impede public health efforts in HIV prevention, testing, treatment, and care.

**Objective:**

The goal of the study is to reduce HIV-related stigma in racialized immigrant communities in 6 Canadian cities in Alberta (Calgary, Edmonton) and Ontario (London, Niagara, Ottawa, Toronto). We will implement an evidence-based 6-session online intervention, Acceptance and Commitment to Empowerment (ACE), to promote skills for psychological flexibility (mindfulness, acceptance, cognitive defusion, values clarification, and committed action) and collective empowerment (critical reflection, dialogue, interconnectedness, and collective action). The objectives are to (1) assess community contexts and needs to inform the refinement of ACE; (2) build community capacity in stigma reduction; (3) evaluate the facilitators and barriers of implementing stigma reduction programs; and (4) disseminate knowledge to advance research, policy, and practice.

**Methods:**

Guided by the principles of social justice and health equity, we will apply the context-based PRISM (Practical Robust Implementation and Sustainability Model) and outcome-based RE-AIM (reach, effectiveness, adoption, implementation, maintenance) frameworks to inform the contextual adaptation, implementation, and evaluation of the ACE intervention. In phase 1 (year 1), we aim to survey 12 local organizations on their existing stigma reduction programs; engage 30 service providers and 60 community members to explore the local contexts and needs of stigma reduction. In phase 2 (year 2), phase 1 results will be used to refine the ACE intervention and apply a train-the-trainer (TTT) approach to engage 48 service providers or community leaders in the ACE training. ACE TTT graduates will be mentored to become ACE Community Facilitators. In phase 3 (years 3‐4), project staff and ACE Community Facilitators will work in small 3-member teams to deliver the online ACE intervention to qualified participants (n=288) in 6 cities. Mixed methods will be used to examine the contextual factors on implementation processes and intervention effectiveness. Data analyses will include reflexive thematic analysis of qualitative data, inferential statistics, and analyses of variance to determine the effectiveness of the intervention over time.

**Results:**

The implementation of project ACE was delayed by 2 years due to the COVID-19 pandemic. The requirement of obtaining research ethics approvals from all affiliated universities (n=8) further delayed the launch of research activities involving participants until September 2023. Between October 2023 and August 2024, the project team recruited 8 community leaders from the 6 project sites to form the Project Advisory Committee (PAC), worked with PAC members to identify outreach and recruitment strategies for phase 1, and recruited and secured 19 community organizations as project collaborators for phase 2. As of March 2025, phase 1 data collection has been completed, and data analysis and knowledge translation are underway.

**Conclusions:**

Based on results from previous research, we anticipate generating critical knowledge on the effectiveness of ACE in reducing internalized and enacted stigma through collective empowerment, and the facilitators/barriers in implementing ACE among racialized immigrants in real-world settings.

## Introduction

Racialized immigrants and refugees are disproportionately impacted by HIV in Canada. Although racialized people constituted 26.6% of the Canadian population as of the 2021 census [1], they continue to be disproportionately impacted by HIV. In 2022, 69.5% (n=519) of first-time HIV cases, where race and/or ethnicity was reported (n=766)**,** were racialized individuals: Indigenous (n=175, 22.6%); Black (n=140, 18%), Asian and Arab (n=126, 16.3%), and Latinx (n=78, 10.1%) [2]. Among Black people in Canada, the proportion of first-time HIV diagnoses across populations increased from 15.6% (n=288) in 2021 to 18.0% (n=140) in 2022, even though they made up 4.3% of the Canadian population [3]. HIV statistics by geographical region show that Ontario maintains the highest number of cases of new HIV diagnoses in Canada. In 2022, for example, Ontario reported 49.1% (n=723) of first-time HIV diagnoses in Canada [3]. Following an increase in the nationwide diagnosis rate in 2023, there was a dramatic increase in new diagnoses in Alberta from 15.6% (n=190) in 2022 to 20.8% (n=255) in 2023, making it the largest increase since HIV was first reported in Alberta in 1998 [3].

Elevated HIV vulnerability for racialized minority groups, including individuals with sexual and gender minority identities, immigrants and refugees, and people without immigrant status, is associated with complex factors, including social isolation [4]; migration/settlement stress [5,6]; systemic barriers to accessing resources [7-9]; and intersecting marginalization related to transphobia/homophobia [10,11], racism [7,12,13], gender-based inequities [4,14], and poverty [15,16]. In addition, HIV stigma and discrimination reinforce HIV vulnerability by creating unsafe environments that deter people from testing and disclosure, resulting in isolation, depression, delayed diagnosis and linkage to treatment and care, and poor health outcomes [17,18]. Stigma also leads to the invisibility of people living with HIV, reinforces community denial, undermines HIV prevention efforts, and impedes provision of accessible community care [19]. However, knowledge gaps in effective and sustainable stigma reduction strategies and programs continue to exist [20]. Further, implementation research that examines effective HIV stigma reduction interventions in real-world settings is also scarce.

**Figure 1.**
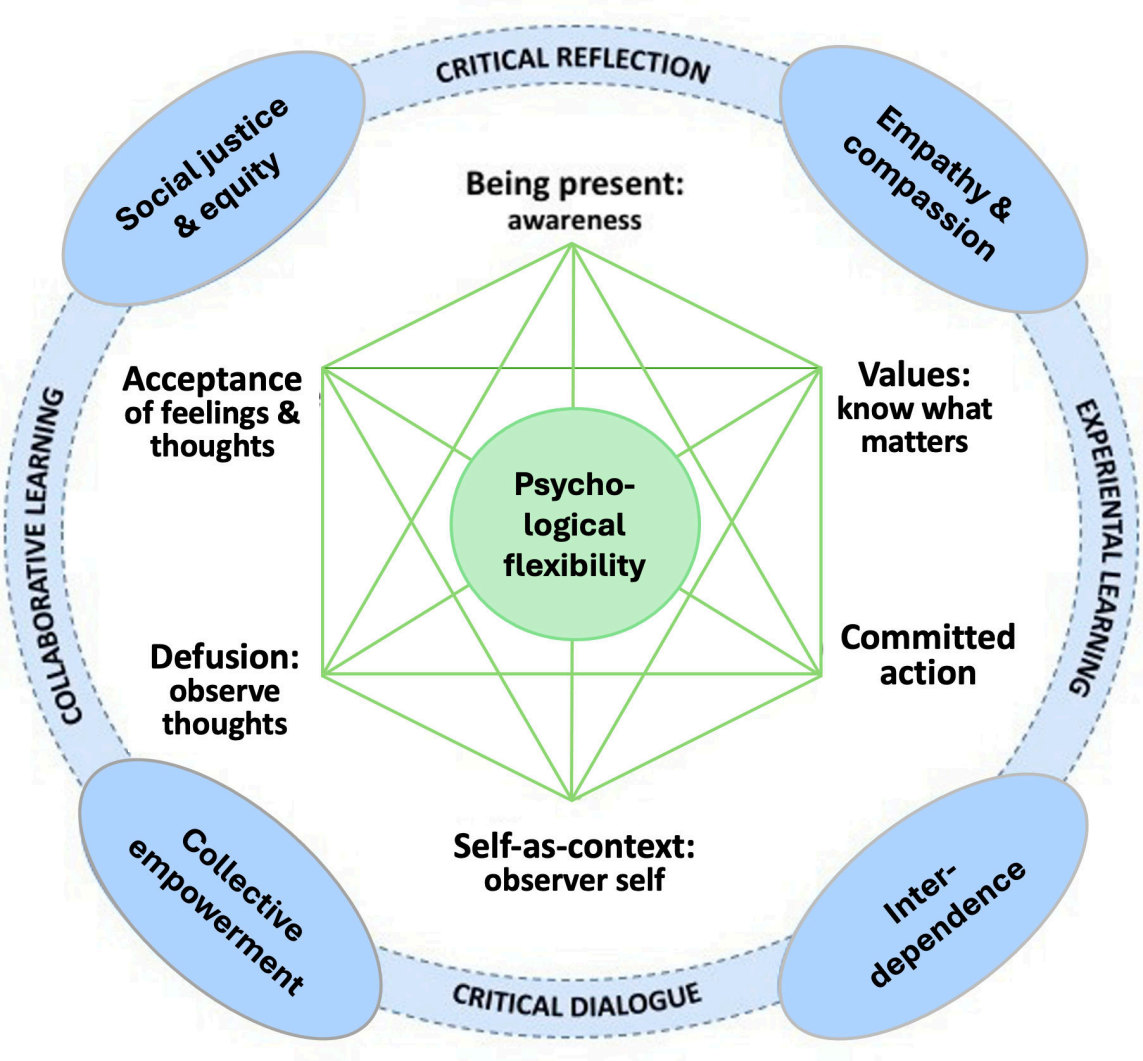
Acceptance and Commitment to Empowerment intervention presented as an integrated model of the acceptance and commitment therapy [24] and the social justice capacity building empowerment model [28].

The Acceptance and Commitment to Empowerment (ACE) intervention is built on existing knowledge and evidence generated from recent research completed by our team [21,22]. HIV stigma is a priority issue that negatively affects people living with HIV or vulnerable to HIV, especially in terms of accessing HIV and mental health care [19,22,23]. Between 2011 and 2015, our team worked closely with Black, Latinx, and Asian communities in Toronto to design and pilot the Community Champions HIV/AIDS Advocates Mobilization Project (CHAMP), funded by the Canadian Institutes of Health Research. The CHAMP study engaged community leaders both living and not living with HIV from faith-based, media, and social justice sectors to address HIV stigma and promote social justice. We evaluated the effectiveness of 2 evidence-based trainings in reducing stigma at the individual and the collective level: (1) acceptance and commitment therapy (ACT) [24,25], an intervention that promotes psychological flexibility through mindfulness-based exercises and experiential activities that are underpinned by 6 core processes: diffusion (observing thoughts as thoughts), acceptance (of experiences of emotions and feelings), contact with the present moment (mindfulness), self-as-context (awareness and self-perspective), values (being clear about what matters), and committed action (based on values) [26]; and (2) social justice capacity building (SJCB) [27,28], an empowerment education that promotes a critical understanding of stigma in the context of power relations in society, recognition of interdependence and interconnectedness as important determinants of health equity, and the importance of tapping into lived experiences and community strengths to take action to reduce stigma. Given their effectiveness, our team integrated ACT and SJCB into a comprehensive model and intervention—ACE, as shown in Figure 1. ACE has been scaled up with service providers and university students in a Global Alliance for Chronic Diseases partnership project in Jinan, China, to reduce mental illness stigma and promote mental health literacy [29,30]. Existing research shows that while online interventions are more costly to develop, well-designed and deployed online interventions can be cost-effective to implement and sustain [31,32].

evieweThe overall goal of this study is to reduce HIV-related stigma in racialized immigrant and refugee communities in 6 Canadian cities in Alberta and Ontario. The objectives are to assess community contexts and needs to inform the refinement of the ACE intervention; build community capacity in stigma reduction using a TTT approach; evaluate the facilitators and barriers of implementing stigma reduction programs; and disseminate knowledge to advance research, policy, and practice. Hence, the overarching research questions are as follows: (1) What are the contextual facilitators and barriers in implementing the evidence-based online ACE intervention in real-world settings? (2) How effective is the online ACE intervention in reducing HIV-related stigma and promoting collective empowerment? Specifically, we will (1) examine the effectiveness of the online training in reducing HIV stigma and promoting collective action; (2) identify the contextual factors contributing to the effective implementation of the online training; (3) determine participants’ characteristics contributing to acceptability and feasibility of the online training; and (4) assess organizational characteristics determining the acceptability and feasibility of the online training.

## Methods

### Conceptual Frameworks

The proposed study is underpinned by the principles of equity, access, and social justice. It is guided by a decolonized critical health promotion framework [33], which centers voices of affected communities and applies strategies such as meaningful engagement [34], shared leadership and responsibilities [35], and capacity building [36] to promote collective empowerment throughout all phases of the research. In addition, we will draw on the PRISM (Practical Robust Implementation and Sustainability Model) and RE-AIM (reach, effectiveness, adoption, implementation, maintenance), well-tested integrated frameworks, to guide the implementation and evaluation of this project. We will apply the RE-AIM framework [37,38] to operationalize the proposed study. The RE-AIM categories will enable us to use consistent methods of evaluation, data collection, analysis, and reporting of results. In addition, the PRISM [39] will enable us to identify and document specific contextual factors that affect implementation at the macro level (eg, public policies, resource distributions, intersecting marginalization, media discourses), meso level (eg, community networks, organizational capacity, practice culture, service user engagement), and micro level (eg, personal values and beliefs, lived experiences). Insights and knowledge of the contextual factors will enable us to identify infrastructures needed for effective implementation and sustainability (eg, policy change, professional development, practice competence, audit-and-feedback loops, and integrated practice, and resources distribution).

**Figure 2.**
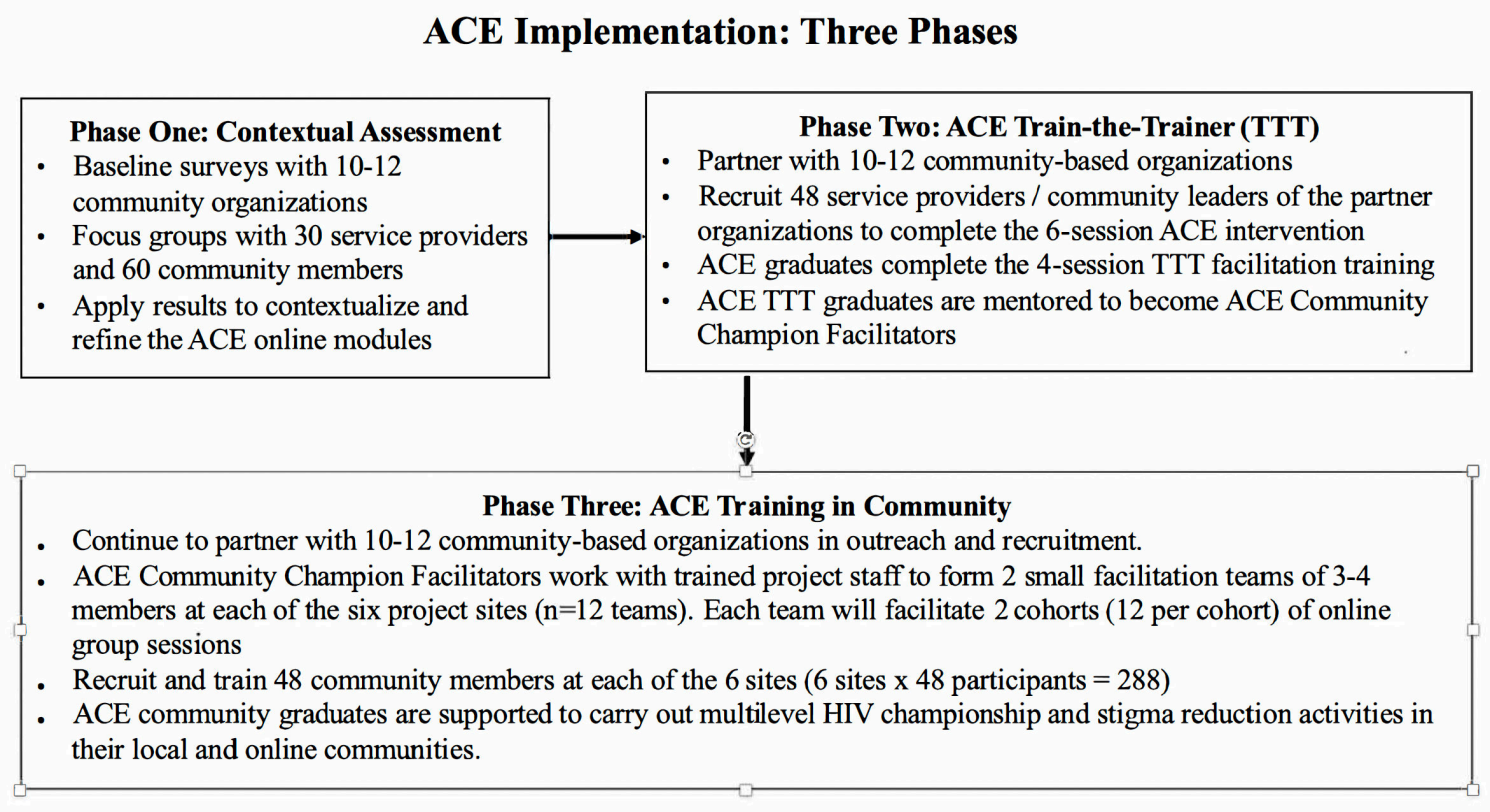
Implementation phases of the Acceptance and Commitment to Empowerment (ACE) intervention.

Project ACE will be implemented in 6 project sites: Calgary and Edmonton in Alberta, and London, Ottawa, Niagara, and the Greater Toronto Area in Ontario. Guided by PRISM and RE-AIM, research activities of community engagement, implementation, evaluation, and knowledge translation and exchange (KTE) will be carried out in 3 phases, as presented in Figure 2.

### Ethical Considerations

The project team has received research ethics approvals from all institutions affiliated with this study. Specifically, the study protocols of the current implementation project have been approved by the research ethics boards of all participating institutions (University of Alberta Pro00127653; University of British Columbia H23-00246; Brock University 22‐122, University of Calgary Pro00127653/pSite-23‐0024; University of Ottawa S-01-23-8683; University of Toronto 43739; Toronto Metropolitan University 2022‐267; Western University 122170; York University e2023-050).

All participants will provide written informed consent before participating in the study. Informed consent will be obtained from all participants before data collection. At each of the research activities, participants will be informed and reminded of the voluntary nature of the research (ie, they can withdraw from the study at any time), and their withdrawals will not affect their relationships with any institutions or service organizations affiliated with this study. The informed consent includes consent for the recording and publication of anonymized information shared during individual participations. Participants will recieve a $30 honorarium as a token of appreciation for their participation in the evaluation survey (i.e., each time they start filling out the pre-survey, post-survey, or 3-month follow-up survey), They will also receive $20 for filling out each of the six online self-directed learning modules, and $10 for each of the three activity logs after the training. Participants will also receive $40 if they are randomly selected and agree to take part in the 3-month post-training focus group.

All research data will be anonymized to protect the privacy or confidentiality of participants. Survey results will be reported in aggregated form without any individual-level identifiers. For participants taking part in the focus groups or group discussions, they may be concerned about their privacy. There may be a risk that their sharing within the focus groups may be shared outside of the group, thus breaching their privacy. The risk will be minimized by (1) informing participants that in a group context, absolute confidentiality cannot be guaranteed and that participants are encouraged to decide what they wish to share and what they do not wish to share; (2) we have included a statement of confidentiality agreement in the participant consent form; and (3) we have also established a set of guiding principles that will be reviewed before all focus groups or group discussions.

### Three Phases of Project ACE

The following section describes the 3 phases of Project ACE. We use a sequential approach in which each phase is informed by the results of the preceding phase.

#### Phase 1 (Year 1): Contextual Assessment

##### Formation of Teams and Project Advisory Committees

In the first 6 months, the team will establish a Project Steering Committee consisting of principal researchers who developed the evidence-based ACE intervention; Regional Research Teams consisting of the coprincipal researchers and coresearchers; and a Project Advisory Committee made up of members including people living with HIV, service providers, community leaders, and collaborators. Project staff will include both people living and not living with HIV drawn from Alberta and Ontario sites. The Project Steering Committee will work with Regional Research Teams to obtain research ethics approval from affiliated universities and organizations.

##### Local Contextual Assessment Specific to HIV Stigma Reduction

###### Group 1: Participating and Collaborating Organizations

We will conduct a 3-month baseline survey with participating organizations to explore the types of services or programs they have offered to address HIV-related stigma and the quantities of services used by their clients and community members (see Multimedia Appendix 1). These data will be used as the baseline to measure reach and effectiveness. Also, we will engage 10 to 12 decision-makers and knowledge users drawn from collaborating organizations every 6 to 9 months in a session of Collaborator Dialogue to share project updates and their perspectives on adopting the ACE intervention to reduce HIV-related stigma, including perceived level of complexity about ACE; organizational values and norms towards new programs or practices; and their perception of potential benefits and challenges in adopting ACE into their organizational practice. Our research team includes 8 community-based organizations as formal collaborators, who will support recruitment of other relevant organizations.

###### Group 2: Service Providers/Community Leaders

We will recruit and engage 5 participants in each of the 6 project sites (n=30) in focus groups to explore their perspectives on the needs of HIV stigma reduction in their organizations and communities, challenges and facilitators of stigma reduction, available resources, and critical aspects of stigma reduction interventions (see Multimedia Appendix 2). Participation criteria will include being aged 18 and older, self-identified as a service provider or community leader working with racially minoritized communities affected by HIV-related stigma in one of the 6 project sites. Prior to the focus groups, participants will be asked to complete a short sociodemographic survey (see Multimedia Appendix 3) to identify the project site they work in, age, sex, gender, ethnicity, first language, religion, and level of community engagement in HIV stigma reduction.

###### Group 3: People Living With HIV And Community Members Affected by HIV-Related Stigma

We will recruit and engage 10 participants in each of the 6 project sites (n=60) in 6 gender-specific and mixed-gender focus groups to explore their experiences and perspectives on HIV stigma, their resistance and resilience strategies, and access to relevant support (see Multimedia Appendix 2 ). Participation criteria will include being aged 18 years and older, self-identifying as belonging to a racially minoritized community, living with and/or affected by HIV-related stigma, and living in one of the 6 project sites. We will pay special attention to recruiting members of diverse sexual and gender identities. Prior to the focus groups, participants will also be asked to complete a short sociodemographic survey (see Multimedia Appendix 3) to identify: the project site they reside in, age, sex, gender, ethnicity, first language, religion, and level of community engagement in HIV stigma reduction.

###### Groups 2 and 3 Recruitment

Participants will be recruited with support from our community collaborating agencies, using common methods of outreach (ie, poster, e-flyers, e-distributions, and social media). Collaborators will be given an electronic copy of the recruitment flyer, which can be printed and posted in their organizations’ public bulletin boards. The e-flyer can be distributed through Twitter, Facebook, collaborators’ e-newsletter, and the project website. This method of distribution has been chosen to ensure that collaborators and partners help to distribute recruitment materials, but they will not do direct recruitment of participants to eliminate any potential conflicts of interest. Direct recruitment will be carried out by project staff. The use of social media will strictly be limited to distribution of our e-flyers; there will be no function for external users to post comments or engage in public communication or interactions. Potential participants will be directed to contact the project coordinator directly, as indicated in the e-flyer.

### Refinement of ACE Intervention

Data on the local contextual assessment will be analyzed, and the results will be used to refine the ACE intervention to best suit the contexts and needs of the participant populations. We will share results from the context assessments with the Local Advisory Committees and our collaborators and engage them in the refinement and adaptation of ACE.

### Phase 2 (Year 2): ACE TTT

#### Overview

We will engage and build capacity among existing service providers and community leaders. We will recruit 8 service providers and community leaders at each of the 6 project sites (n=48) in the HIV and related sectors (eg, mental health, addiction, sexual health, settlement services). We will apply a TTT capacity-building approach to implement and evaluate the effectiveness of the ACE intervention in reducing stigma and motivating the ACE graduates to become cofacilitators to implement ACE in the community in phase 3. Phase 2 participation criteria include being aged 18 years and older, self-identifying as a service provider or community leader working with racially minoritized communities affected by HIV-related stigma in one of the 6 project sites, being available and committed to complete the ACE intervention and become an ACE Champion to co-facilitate the ACE intervention in their local communities, and having fluency in written and spoken English. Phase 2 learning activities include the following: pretraining self-directed learning (“Stigma Reduction 101”), the ACE intervention, TTT practice workshops, and posttraining support.

#### Pretraining Self-Directed Learning (“Stigma Reduction 101”)

Participants will complete an online preintervention survey and one 30-minute online module called HIV Stigma 101, hosted on a Learning Management System. The HIV Stigma 101 will be developed by the team, drawing on the results of our Contextual Analysis in phase 1, as well as culturally safe and up-to-date HIV information on the HIV care cascade.

#### The ACE Intervention

A 6-week online experiential learning activities to (1) promote participants’ psychological flexibility; (2) reduce HIV-related stigma; (3) promote resilience/effective coping; and (4) increase individual and collective empowerment that enhance community championship. The online training consists of 6 weekly 60-minute self-directed interactive online learning modules, which will be reinforced by a 1.5-hour weekly videoconferencing session. Each module will cover one of the 6 ACT processes along with their corresponding SJCB principles and processes. Our proposed online ACE intervention is innovative in that, unlike many online psychological interventions that offer individual self-directed learning [40,41], it includes group collaborative learning through videoconferencing to promote engagement and collective empowerment.

#### TTT Practice Workshops

The ACE TTT graduates will receive the ACE Intervention TTT Manual and access to training videos. They will also take part in four online 2.5-hour TTT workshops to learn about: outreach and recruitment; data collection; the ACE concepts in detail; online facilitation and debriefing of the 6 online sessions; and practice facilitation with peer and mentor feedback.

#### Posttraining Support

We will develop a community of practice utilizing the Learning Management System to engage the ACE TTT graduates and support them in taking on the role as ACE Community Facilitators. They will be engaged in asynchronous and scheduled online structured peer-sharing and periodic mentorship sessions during the 6-month practicum period to enact phase 3 (ie, ACE facilitation in the community). They will be encouraged to engage in regular reflections post-training on the impact of ACE on their personal, professional, and community lives and submit an online activity log of their championship activities.

### Phase 3 (Years 2-4): ACE Community Champions in Action

The ACE Community Facilitators will be “mobilized” to implement HIV-related stigma reduction activities, including the following: recruitment, delivery of the ACE intervention, and stigma reduction championship.

#### Recruitment

The ACE Community Facilitators at each site (n=8) will be divided into 2 teams of 3 to 4 members (accounting for job change and attrition). With the support of their organizations, each team will work with Project ACE staff to recruit 24 service users and/or community members as participants (6 sites × 2 teams × 24 persons; n=288). Since Project ACE is an implementation study that focuses on identifying the facilitators and barriers to implementing evidence-based practice in real-world settings, we will not use any control groups. We will pay special attention to recruiting members of diverse sexual and gender identities.

Phase 3 participation criteria for the community ACE intervention includes: aged 18 years and older, living in one of the 6 project sites, self-identifying as belonging to a racially minoritized community and a priority group affected by HIV stigma and/or identified by the partner organizations (eg, LGBTQ+ [lesbian, gay, bisexual, transgender, queer, and other gender and sexual minorities], newcomers, youth, women), is available and committed to attending all data collection and training sessions and interested in becoming an HIV community champion to reduce HIV-related stigma (eg, substance use, racism, transphobia) in the community.

#### Delivery of the ACE Intervention

Each ACE Community Facilitator team will deliver 2 cohorts of online ACE facilitation. The aim of the ACE intervention is to reduce HIV-related stigma and promote resilience among the participants. Graduates of the community ACE intervention training will become ACE community champions. In phase 3, we will use the same online ACE intervention used in phase 2, consisting of 6 weekly modules of 60-minute self-directed interactive online learning activities reinforced by a 1.5-hour weekly videoconferencing session. Our proposed online ACE intervention is innovative in that, unlike many online psychological interventions that offer individual self-directed learning only [40,41], it includes group collaborative learning through videoconferencing to promote peer support, community engagement, and collective empowerment.

#### Stigma Reduction Championship

The ACE Intervention Community Facilitators will continue to engage in the community of practice to consolidate their ACE knowledge and skills and contribute to community-wide stigma reduction by connecting the newly trained ACE graduates (n=288) to local organizations, groups, or initiatives, and providing them with support in carrying out HIV-related stigma reduction activities (eg, peer workshops, media interviews, newsletters). ACE graduates will be invited to submit 3 monthly community champion activity logs that capture these activities.

### Evaluation of ACE Intervention (Years 2-4)

We will use mixed methods to collect data, including surveys with sociodemographic questions (see Multimedia Appendix 3) and validated scales (listed below), focus groups, dialogical sessions, document analysis, field observation, and tracking of activities of interest. We will use PRISM [39] to contextualize and RE-AIM [37,38] outcomes in evaluating the implementation of the ACE intervention.

#### Reach

We will measure the absolute number, proportion, and representativeness of the intended population willing to, or taking part in the ACE intervention. The reach of the ACE intervention will be the proportion of service providers, community leaders, service users, and community members recruited and successfully trained compared to the estimated corresponding eligible populations. As we have set a predetermined recruitment target (8 TTT participants per site; 48 community participants per site) and composition (eg, service providers/community leaders, people affected by HIV stigma), we will keep track of those who expressed interest to be trained beyond the target as an estimate of “maximal reach.” The total number of eligible participants will be obtained based on information from the local advisory committees. We will keep track of noncompleters and examine for any significant differences in demographics and other baseline variables between completers and noncompleters, as well as between completers and known data of the populations in each site.

#### Effectiveness

This measures the impact of the ACE intervention on our intended outcomes (ie, stigma reduction and collective empowerment). We will evaluate the effectiveness of the ACE intervention with corresponding validated measures at preintervention, immediate postintervention, and 3-month postintervention. In addition to a short sociodemographic survey on the project site participants work in or reside in, age, sex, gender, ethnicity, first language, religion, and level of community engagement in HIV stigma reduction, relevant validated scales to be used include (1) the 12-item HIV Stigma Scale [42], which include four subscales on personalized stigma, disclosure concerns, concerns with public attitudes and negative self-image, (2) the 4-item Perceived Discrimination from Service Providers Scale [43] with dichotomized score (yes/no) to measure perceived HIV-related discrimination from health care providers, (3) the 12-item Cognitive and Affective Mindfulness Scale (CAMS-R) [44,45] that assesses mindfulness using language that is not connected to any specific style of meditation practices, (4) the 7-item Acceptance and Action Questionnaire Version II (AAQ-II) [46] that measures experiential psychological inflexibility and cognitive fusion, (5) the 28-item Brief Coping Orientation to Problems Experienced (Brief COPE) [47] that measures 14 different coping strategies (self-distraction, active coping, denial, substance use, use of emotional support, use of instrumental support, behavioral disengagement, venting, positive reframing, planning, humor, acceptance, religion, and self-blame); and (6) the 12-item Self-Compassion Scale Short (SCS-S) [48] that measures self-kindness, self-judgment, common humanity, isolation, mindfulness, and over-identification. Focus groups (see Multimedia Appendix 4) will also be conducted at 3-month post-training to explore (1) participants’ experiences of taking part in the ACE intervention; (2) how participants have applied what they learned in ACE in their everyday life to address stigma; and (3) how ACE can be improved.

#### Adoption

This component measures the absolute number, proportion, and representativeness of organizations willing to participate in this project. The adoption of the ACE intervention, or the support of organizations/settings to implement ACE, will be assessed in terms of perceived feasibility, acceptability, and fit based on results from the local Contextual Assessment (phase 1). We will also track expressed interest and diffusion attributes by stakeholders in the semiannual dialogical sessions, contextual factors that facilitate or impede adoption/adaptation, and the potential and actual uptake of the ACE intervention by organizations within or outside of the HIV/sexual health sector in each site, and in other cities in Alberta and Ontario.

#### Implementation

We will assess the extent to which the intervention is delivered as intended. To achieve this, fidelity checklists will be used to evaluate the implementation of the ACE intervention by the staff facilitators and the trained ACE Intervention Community Facilitators. Participants will complete a postsession feedback form at each training session to assess their self-report of satisfaction, knowledge gained, and confidence in specific skills. ACE facilitators will complete a debriefing form after each training session to document implementation issues and group dynamics. Finally, we will conduct a cost analysis on community-engaged implementation [49] of the ACE intervention.

#### Maintenance

We will measure maintenance and sustainability of the ACE intervention at both the individual and organizational levels over the period of follow-up, especially with a focus on activities that can be sustained beyond the project, and diffusion networks (ie, potential uptake by organizations and interest holders not directly involved in the study). This includes activities by ACE Champions and community participants captured in monthly activity logs, the online responses in the Learning Management System, and community requests for ACE intervention. In addition, an online qualitative survey will be conducted 3 months postintervention with participants and interest holders to evaluate: the impact of ACE at the personal, professional, organizational, and community levels, acceptability of ACE, desire for adoption, appropriateness, and feasibility.

### Data Analysis (Years 1-4)

#### Overview

In addition to using the RE-AIM Framework, we will use a multiple-case study design to examine the contextual factors that support or hinder effective implementation of ACE in the six project sites. The case study approach is suitable for implementation research because it allows for an in-depth investigation of real-life phenomena within contexts (eg, an individual, a group, an organization, a community, an event, or a problem) [50,51]. In case study research, the contextual conditions are not controlled but considered part of the investigation. The multiple case design allows us to perform within-case (eg, online ACE intervention, Black women, or the Calgary site) as well as across-case analyses (eg, comparison of effectiveness of site-specific intervention groups, implementation facilitators, and barriers across all six sites, or across different ethno-cultural communities). We will apply triangulation as a data collection and analytic strategy to achieve detailed case descriptions and to gain a critical understanding of “how” and “why” things happen [52,53]. We will also apply cross-case analysis to identify similarities and differences in the factors, conditions, and contexts that support or hinder effective implementation of the ACE intervention in the 6 sites.

#### Quantitative Data Analysis

Descriptive and group-based analyses will be conducted to gain insights into the sampled population. Inferential statistics, including analyses of variance, will be conducted to determine the effectiveness of the interventions over time in terms of their contribution to decreasing stigma (stigma scales); increasing psychological flexibility (AAQ-II); increasing mindfulness (CAMS-R); increasing self-compassion (SCS-S); and decreasing negative coping (Brief Cope). Regression, path analyses, and hierarchical structural equation modeling will be conducted to examine the potential mediating and moderating impact of other variables, such as background demographics, level of participation, roles in community (service provider, leader, service user), and involvement in HIV-AIDS championship activities, on project and intervention outcomes.

#### Qualitative Data Analysis

Focus group interviews (see Multimedia Appendix 4) will be transcribed verbatim. A computer software program (N-Vivo) will be used to aid in data management. For data analysis, we will use both inductive and deductive approaches to manually look for broad categories that were *voice-centered* articulated by the participants (inductive) and *sensitizing* drawn from pre-existing theories and the research questions (deductive) in the transcripts [54]. Guided by a decolonized critical health promotion framework [33] and using an iterative and systematic approach [55-57], we will (1) familiarize ourselves with the data; (2) review our focus group discussion guide and the PRISM/RE-AIM framework; (3) generate codes as the smallest units of analysis responsive to the research questions [58]; (4) develop a coding tree with subcodes to facilitate coding for analysis and interpretation; (5) make sense of and use codes as building blocks to generate themes as central organizing concepts [57,59]; (6) engage team members in reflexive analysis and interpretation; and (7) produce reports and relevant knowledge products based on the results. These iterative team processes will ensure that collective reflexive insights are integrated into the data analysis and consensus is reached in understanding the data.

## Results

Although Project ACE was funded in October 2020, project implementation was delayed by almost 2 years due to the impact of the COVID-19 pandemic. In June 2022, research preparation activities were launched; they included the establishment of a Project Steering Committee, hiring and training of project staff, and the development of the online training platform. However, the requirement of receiving research ethics approvals from all affiliated universities (n=8) further delayed the launch of research activities involving participants until September 2023. Between October 2023 and August 2024, the project team recruited 8 community leaders from the 6 project sites to form the Project Advisory Committee (PAC), worked with PAC members to identify outreach and recruitment strategies for phase 1, recruited and secured 19 community organizations as project collaborators for phase 2. As of March 2025, 18 organizations have completed the baseline survey, and 32 service providers and 53 community members have been successfully recruited in the phase 1 contextual assessment. Data analysis is underway. A community infographic report on phase 1 is expected to be published in February 2026, and 3 peer-reviewed manuscripts are expected to be published by August 2026.

## Discussion

Drawing on the results of our previous research, we anticipate that Project ACE will contribute critical new knowledge to inform stigma reduction and effective implementation. Although HIV stigma occurs in multiple levels (intrapersonal, interpersonal, organizational, community, and societal), most stigma reduction interventions only address stigma at the personal or interpersonal levels [60]. Building on a decade of stigma reduction research [19,22], our team has developed the ACE intervention, which addresses stigma through skills building to increase psychological flexibility and readiness for value-guided committed action at multiple levels [61].

In this project, we are drawing on a decolonized critical health promotion framework [33] and the well-established PRISM/RE-AIM implementation evaluation framework [37-39]. The integrated use of these frameworks places us in a strong position to identify, at multiple levels, factors that perpetuate HIV stigma and the contextual facilitators and barriers in implementing antistigma intervention in real-world settings. For example, in our previous research, the application of a critical health promotion framework enabled us to challenge the popular perception of stigma as individual behaviors. We documented how structural and system determinants, such as homophobia, racism, gender-based discrimination, and unequal power relations, interacted to produce and perpetuate stigma and misconceptions that deterred community leaders from engaging in collective action to reduce stigma [19].

Our innovative pre-post-follow-up mixed methods design also positions our team to generate important knowledge. Data collection will include sociodemographic surveys, validated scales, focus groups, and meeting notes. In addition, all ACE participants will be invited to complete regular activity logs for 3 months postintervention. The use of activity logs will enable us to measure not only changes in participants’ attitude, emotional regulation, degree of coping, and sense of readiness to engage in collective action on HIV stigma, but we will be able to document their actual behavioral changes at personal, interpersonal, organizational, and community levels. For example, we used activity logs for data collection in our CHAMP study, in which we recruited and engaged 104 participants, and 62 completed a similar intervention. These 62 graduates carried out 1090 multilevel stigma reduction and equity-based activities over 9 months postintervention. The champion activities included providing peer support, volunteering at an HIV/AIDS organization, leading health literacy workshops, establishing new social groups, and confronting homophobia at a faith-based organization [62].

Similarly, the Linking Hearts project engaged 146 multidisciplinary professionals at 6 universities in China to promote student mental health and reduce stigma. A total of 124 participants completed the in-person ACE-LYNX-PRO (Acceptance and Commitment to Empowerment–Linking Youth and “Xin” (hearts) with Professionals) intervention, online pre-post-follow-up surveys with validated scales, 3-month postintervention focus groups, and weekly online activity logs for 3 months postintervention, yielding 1083 entries. Survey results showed statistically significant psychological empowerment measured through reduction in stress and in stigmatizing attitude. Analysis of the activity logs and focus groups showed committed action at multiple levels: (1) individual-level activities included counseling individuals, self and family care, building personal skills; (2) group-level activities included structured ACE-based group counseling; facilitating peer dialogue on mental health; engaging in collaborative learning; (3) organizational-level activities included initiating psychoeducation and mental health advocacy; integrating ACE into mental health education curricula; coordinating mental health awareness campaigns; establishing book clubs; and (4) community-level activities included ACE-based experiential workshops and mental health literacy promotion among neighborhood residents [61].

Based on previous research results, it is anticipated that participants in Project ACE will experience psychological empowerment and demonstrate committed action in HIV championship and stigma reduction at multiple levels. At the same time, we also anticipate some potential limitations. Since this is an implementation study that demands a lengthy commitment from participants, the study withdrawal rates may be high. We are addressing this potential challenge using intentional over-recruitment. In addition, the extent of successful implementation of the ACE intervention is dependent on many social, political, and economic contextual factors at the organizational, community, and societal levels that are beyond the control of our team. However, as an implementation study, our meticulous systematic documentation and rigorous analysis will enable us to generate knowledge that is informative and pragmatic.

The research team is strongly committed to creating knowledge and putting knowledge into action. An integrated knowledge translation approach will be applied to engage community members, service providers, and decision makers throughout all phases of the study. This will include (1) regular updates and Knowledge Translation involving posting and circulation of three newsletters per year to disseminate research updates and to promote community engagement. Also, semi-annual dialogical sessions will be held every 6 to 9 months to promote evidence uptake and ACE integration into their programs and services; (2) participant-driven KTE through collaboration with our ACE champions to deliver a variety of KTE activities to reduce stigma within their communities (eg, media interviews, presentations at faith-based events, digital stories, graphic novels, educational forums); and (3) end-of-grant KTE through collaboration with people living with HIV, ACE champions, collaborators, service providers, community leaders, health policy/decision-makers, and researchers. The collaborative team will produce policy briefs, media reports, infographic factsheets, community reports, digital stories, graphic novels, peer-reviewed conference presentations, and peer-reviewed publications. Finally, a KTE summit will be held to bring together different interest-holders to dialogue and advance policy, practice, and action.

## Supplementary material

10.2196/80669 Multimedia Appendix 1 Phase 1 contextual assessment: collaborator baseline survey.

10.2196/80669 Multimedia Appendix 2 Phase 1 Acceptance and Commitment to Empowerment (ACE) contextual assessment: focus group discussion guides.

10.2196/80669 Multimedia Appendix 3 Participant sociodemographic survey (all phases).

10.2196/80669 Multimedia Appendix 4 Phases 2 and 3 postintervention focus group discussion guide.

10.2196/80669 Peer Review Report 1 Peer review report by Psychosocial, Sociocultural & Behavioral Determinants of Health Review Committee, Canadian Institutes for Health Research (CIHR).

## Abbreviations

AAQ-II: Acceptance and Action Questionnaire Version II
ACE: Acceptance and Commitment to Empowerment
ACE-LYNX-PRO: Acceptance and Commitment to Empowerment–Linking Youth and ‘Xin’ (hearts) with Professionals
ACT: acceptance and commitment therapy
Brief COPE: Brief Coping Orientation to Problems Experienced
CAMS-R: Cognitive and Affective Mindfulness Scale
CHAMP: Community Champions HIV/AIDS Advocates Mobilization Project
KTE: knowledge translation and exchange
LGBTQ+: lesbian, gay, bisexual, transgender, queer, and other gender and sexual minorities
PRISM: Practical Robust Implementation and Sustainability Model
RE-AIM: reach, effectiveness, adoption, implementation, maintenance
SCS-S: Self-Compassion Scale Short
SJCB: social justice capacity building
TTT: train-the-trainer
