# Supplementary material for: Acceptance and Commitment to Empowerment Intervention to Reduce HIV Stigma and Promote Community Resilience: Protocol for an Implementation Study
Source: JMIR Res Protoc. 2026 Jan 26;15:e80669. doi: 10.2196/80669 (PMC12834450; doi:10.2196/80669)
Supplement: Multimedia Appendix 1 [file resprot-v15-e80669-s001.pdf]

Date: ________________

Project site: Please check One

☐	Calgary		☐	London
☐	Edmonton		☐	Niagara
☐	Greater Toronto Area		☐	Ottawa

Baseline of Services (Collaborating Organizations)

1. Which of the following areas does your organization provide services in?

   ☐ Primary care
   ☐ Legal services
   ☐ Mental health
   ☐ Social services
   ☐ Faith based services
   ☐ HIV / Sexual health
   ☐ Settlement
   ☐ Addiction
   ☐ Peer support / Mutual support groups or networks
   ☐ Other, please specify __________________

2. How many people access the programs and services at your organization per year?
   ____

3. In the past 3 months, has your organization provided services to racialized immigrants and/or refugees?

   ☐ Yes ☐ No ☐ Don’t know

   If yes, how many percent of your service users were racialized immigrants/refugees?
   ____

4. In the past 3 months, has your organization provided services to people living with HIV?
   ☐ Yes ☐ No ☐ Don’t know

   If yes, how many percent of your service users were people living HIV? ________________

5. In the past 3 months, has your organization provided services to people living with addiction or substance use?
   ☐ Yes ☐ No ☐ Don’t know

   If yes, how many percent of your service users were people living with substance use or addiction challenges? ________

6. In the past 3 months, has your organization provided services to people living with mental illness?
☐ Yes ☐ No ☐ Don't know

If yes, how many percent of your service users were people living with mental illness? ______

7. In the past 3 months, has your organization provided services to someone engaging in sex work?
☐ Yes ☐ No ☐ Don't know

If yes, how many percent of your service users were someone engaging in sex work? ______

8. Does your organization have written guidelines to protect the following groups of people from discrimination?

**Statements**	Yes	No	Don't know
Racialized immigrants / refugees			
People living with HIV			
People living with addiction			
People living with mental illness			
People engaging in sex work			

9. Has your organization provided all staff with training on the following topics?

**Statements**	Yes	No	Don't know
Racism and racialized stigma			
Stigma of HIV			
Stigma of addiction/substance use			
Stigma of mental illness			
Stigma of sex work			

10. In the past 3 months, has your organization provided any programs or services to address the following stigmas?

**Statements**	Yes	No	Don't know
Racism and racialized stigma			
Stigma of HIV			
Stigma of addiction/substance use			
Stigma of mental illness			
Stigma of sex work			

11. Additional comments:

____

____
