# Supplementary material for: Acceptance and Commitment to Empowerment Intervention to Reduce HIV Stigma and Promote Community Resilience: Protocol for an Implementation Study
Source: JMIR Res Protoc. 2026 Jan 26;15:e80669. doi: 10.2196/80669 (PMC12834450; doi:10.2196/80669)
Supplement: Multimedia Appendix 2 [file resprot-v15-e80669-s002.pdf]

PART A -- Participant Group: Service Providers / Community Leaders

1. Introduction

- Welcome participants; review research information; answer questions; confirm informed consent; review guidelines on confidentiality and respect.
- Facilitators explain the purpose of contextual assessment and provide a preamble on how stigma affects community HIV responses and the health of people living with or affected by HIV.

2. Explore participants' perspectives on HIV related stigma

You have been invited to participate in this study because you are a service provider / community leader working with individuals and groups living with, affected by, or are vulnerable to HIV. We would like to hear your perspectives about the key challenges related to HIV stigma faced by your service users or members in your community.

- Probe: explore how stigma affects different groups differently --
    - gender (cisgender women vs. men; transgender and gender queer people, etc.); sexualities; substance use; sex work, etc.

3. Explore participants' experience in addressing and/coping with HIV related stigma

- When you experienced stigma in the past, how did you deal with it?
    - Probe: explore types of stigma, coping strategies, personal and community resources, impact on health and wellbeing.

4. Explore participants' perceptions of community values and attitude on HIV related issues

- What are the values and beliefs in the communities you work with that support people who are HIV+ and/or vulnerable to HIV?
- What are the values and beliefs that get in the way of supporting people who are HIV+ and/or vulnerable to HIV?
    - Probe: values across different communities – faith-based, ethnocultural, etc.

5. Explore participants' Perspectives about current HIV related stigma reduction strategies within their communities

- In the communities that you work with or serve, what kinds of initiatives or activities are there to reduce HIV related stigma?
    - Probe: initiatives that are raising awareness of HIV; anti-homophobia initiatives; harm reduction initiatives, etc.
- What are your thoughts about these initiatives?
    - Probe: explore questions -- What works? What does not work? What needs to happen to improve these initiatives?
- What is missing, or what kinds of initiatives are needed to reduce HIV related stigma?
    - Probe: explore -- what needs to happen to address the needs?

6. Additional thoughts or comments

PART B -- Participant Group: Community Members/Service Users

1. Introduction

- Welcome participants; review research information; answer questions; confirm informed consent; review guidelines on confidentiality and respect.
- Facilitators explain the purpose of contextual assessment and provide a preamble on how stigma affects community HIV responses and the health of people living with or affected by HIV.

2. Explore participants' perspectives on HIV related stigma

- We would like to hear your perspectives about the key challenges related to HIV stigma faced by you and/or your peers.
- Probe: explore how stigma affects different groups
  - gender (cisgender women vs. men; transgender and gender queer people, etc.); sexualities; substance use; sex work, etc.

3. Explore participants' experience in addressing and/coping with HIV related stigma

- When you experience stigma in the past, how did you deal with it?
  - Probe: explore types of stigma, coping strategies, personal and community resources, impact on health and wellbeing, etc.

4. Explore participants' perceptions of community values and attitude on HIV related issues

- What are the values and beliefs in the communities you work with that support people who are HIV+ and/or vulnerable to HIV?
- What are the values and beliefs that get in the way of supporting people who are HIV+ and/or vulnerable to HIV?
  - Probe: values across different communities – faith-based, ethnocultural, etc.

5. Explore participants' perspectives about current HIV related stigma reduction strategies within their communities

- In the communities that you identify with, what kinds of initiatives or activities are there to reduce HIV related stigma?
  - Probe: initiatives that are raising awareness of HIV; anti-homophobia initiatives; harm reduction initiatives, etc.
- What are your thoughts about these initiatives?
  - Probe: explore -- What works? What does not work? What needs to happen to improve these initiatives?
- What is missing, or what kinds of initiatives are needed to reduce HIV related stigma?
  - Probe: explore what needs to happen to address the needs.

5. Additional thoughts or comments
