# Supplementary material for: Acceptance and Commitment to Empowerment Intervention to Reduce HIV Stigma and Promote Community Resilience: Protocol for an Implementation Study
Source: JMIR Res Protoc. 2026 Jan 26;15:e80669. doi: 10.2196/80669 (PMC12834450; doi:10.2196/80669)
Supplement: Multimedia Appendix 3 [file resprot-v15-e80669-s003.pdf]

Date: _________________

Project site: Please check One

☐	Calgary	☐	London
☐	Edmonton	☐	Niagara
☐	Greater Toronto Area	☐	Ottawa

Section 1: Demographic and Health Service Access Information

1. What sex were you assigned at birth?
   - ☐ Female
   - ☐ Male
   - ☐ Intersex
   - ☐ Don't know
   - ☐ Option not listed, please specify _________________

2. What gender do you currently identify with?
   - ☐ Woman
   - ☐ Man
   - ☐ Transman (assigned female at birth, identify as a man)
   - ☐ Transwoman (assigned male at birth, identify as a woman)
   - ☐ Non-binary
   - ☐ Two-spirit
   - ☐ Option not listed, please specify ____________________

3. How would you describe your sexual orientation?
   - ☐ Heterosexual / Straight
   - ☐ Gay
   - ☐ Lesbian
   - ☐ Bi-sexual
   - ☐ Two-spirit
   - ☐ Questioning / Unsure
   - ☐ Option not listed, please specify _____________

4. What year were you born? ______________________

5. Where (name of country) were you born? __________________________________

6. If you were not born in Canada, how long have you lived in Canada? ______________________

7. What is your first language? _______________________

8. Which of the following best describes your religion / faith / spirituality? (Please check all that apply.)

☐ Buddhist
☐ Catholic
☐ Hindu
☐ Indigenous spirituality
☐ Jewish
☐ Muslim
☐ Protestant
☐ Sikh
☐ No religious affiliation
☐ Option not listed, please specify ____________________

9. Which of the following best describes your ethno-racial identity?

☐ Indigenous/Aboriginal (First Nations / Metis / Inuit)
☐ African / Caribbean / Black
☐ White / European (e.g., British, French, German, Russian, etc.)
☐ East or Southeast Asian (e.g., Chinese, Filipino, Korean, Japanese, etc.)
☐ South Asian (e.g., East Indian, Pakistani, Sri Lankan, etc.)
☐ Latin American / Hispanic (e.g., Mexican, Brazilian, El Salvadorian, etc.)
☐ West Asian / Middle Eastern / North African (e.g., Iranian, Egyptian, Arab, etc.)
☐ Option not listed, please specify ____

10. Which of the following groups do you identify with? (Please check all that apply.)

☐ Partner / Caregiver of PLHIV
☐ Sexual minority people
☐ Immigrant, refugee, non-status people
☐ People living with HIV
☐ People living with Hepatitis C
☐ People with mental health challenges
☐ People using drugs
☐ People with addiction challenges
☐ Option not listed, please specify ___________________

11. For the group that you identify to be your closest community (can be from the lists above or other ones), how satisfied are you with your current level of involvement or connection to them?

Group or community: ____

☐ Completely satisfied
☐ Somewhat satisfied
☐ Somewhat not satisfied
☐ Completely not satisfied

12. Are you a **service provider**?

☐ No
☐ Yes

If yes, which of the following areas do you provide services in?

☐ Primary care
☐ HIV / Sexual health
☐ Legal services
☐ Settlement

☐ Mental health ☐ Addiction / Harm reduction
☐ Social services ☐ Peer support / Mutual support groups or networks
☐ Faith based services ☐ Option not listed, please specify __________________

13. Are you a **<u>service user</u>**?

☐ No ☐ Yes

If yes, at which type of organizations have you received services in the last 3 months? (Check all that applies)

☐ Primary care ☐ HIV / Sexual health
☐ Legal services ☐ Settlement services
☐ Mental health services ☐ Addiction/Harm reduction services
☐ Social services ☐ Peer support / Mutual Support groups or networks
☐ Faith-based services ☐ Option not listed, please specify __________________

13. Thinking about your current activities in providing service / volunteering / community engagement, what is the percentage of your current total time spent on **activities to reduce stigma related to HIV**?

☐ 0% - 10% ☐ 11% - 20%
☐ 21% - 30% ☐ 31% - 40%
☐ 41% - 50% ☐ 51% - 60%
☐ 61% - 70% ☐ 71% - 80%
☐ 81% - 90% ☐ 91% - 100%
