# Supplementary material for: Acceptance and Commitment to Empowerment Intervention to Reduce HIV Stigma and Promote Community Resilience: Protocol for an Implementation Study
Source: JMIR Res Protoc. 2026 Jan 26;15:e80669. doi: 10.2196/80669 (PMC12834450; doi:10.2196/80669)
Supplement: Multimedia Appendix 4 [file resprot-v15-e80669-s004.pdf]

1. **Introduction:**
    - Welcome participants; review purpose of focus group; answer questions; review guidelines on confidentiality and respect.
    - Review the topics covered in the ACE training program – put in zoom chat or post as a slide of ppt

2. **Explore participants' experiences in participating in the ACE training:**
    - What was your overall experience like?
    - In terms of learning approaches, what did you like the most? What did you like the least?
        - Probe: experiential, individual vs. group, mindfulness, etc.
    - In terms of contents of the training, what did you find most useful? What did you find least useful?
        - Probe – in stigma reduction (HIV, substance use, sexism, homophobia, transphobia, racism, etc.)
        - Probe – in psychological acceptance and flexibility
        - Probe – readiness to take action to reduce stigma

3. Since you completed the ACE training, how have you been able to apply what you have learned in your everyday life?

- In your personal life/personally
    - Probe – self-care, health practices, stress reduction, increased use of social support
- In your professional life (for service providers / leaders only)
    - Probe – applying it in everyday work, using it with service users
- In your social networks - among family, friends, co-workers, etc.
    - Probe – share stress reduction skills, address HIV and related stigmas
- In the community – ethnocultural, or LGBTQ, or service provider associations, etc.
    - Probe – increased participation in community activities; address HIV and related stigmas, etc.

4. How can the ACE training program be improved?

Probe: length, contents, approaches, other communities

5. Invite participants for additional comments.
