# Supplementary material for: Acceptance and Commitment to Empowerment Intervention to Reduce HIV Stigma and Promote Community Resilience: Protocol for an Implementation Study
Source: JMIR Res Protoc. 2026 Jan 26;15:e80669. doi: 10.2196/80669 (PMC12834450; doi:10.2196/80669)
Supplement: Peer Review Report 1 [file resprot-v15-e80669-s005.pdf]

**Review Type/Type d'évaluation:** SO Notes /Notes de l'agent scientifique
**Name of Applicant/Nom du chercheur:** Wong, Josephine P.H.
**Application No./Numéro de demande:** 437709
**Agency/Agence:** CIHR/IRSC
**Competition/Concours:** 2020-03-04 Project Grant/Subvention Projet
**Committee/Comité:** Psychosocial, Sociocultural & Behavioural Determinants of Health/Déterminants psychosociaux, socioculturels et comportementaux de la santé
**Title/Titre:** Acceptance and Commitment to Empowerment (ACE) Intervention: Reducing HIV Stigma and Promoting Community Resilience Through Capacity Building

---

**Assessment/Évaluation:**

The committee were enthused about this potentially impactful and very innovative and participatory mixed method project.

**Strengths**
It was seen to be a natural next ‘scaling’ step for this productive research group, involved appropriate and clearly impeccably well engaged community partners and addresses a range of important health issue in Canada. The PI is an international leader in this area.
The proposal was seen to be well grounded in the social justice movement and relevant pilot work.
The complex project was well organized and the KT plan was strongly detailed, comprehensive and appropriate.
Sex and gender, in particular, were well handled throughout the grant with thoughtfulness and nuance.
Weaknesses noted by reviewers were comparatively minor.
**Weaknesses**
The project is ambitious and complex – and handling this better would improve the project.
Differentiation between the training and intervention pieces was not always clear. Better definitions of training and intervention and differentiation of these aspects may help. This raised confusion at times about the procedures and outcomes of the grant (for example who is involved ,when) – and what success would look like. Better organization of the writing may help this.
The feasibility of the project in the context of the complexity was raised.
The qualitative element was at times to general.
More linkages to justify the ACE framework benefits could have been made, more detail on the selection of research sites could have been included. More details on the expected post training reflections and quantitative analyses, and the collaborative data collection would have been useful.
Other concerns were noted about how the analyses would explore the influence of sex and gender could have been included.
These concerns were however minor in the context of this timely and complex work.
